# Establishing reference ranges for circulating biomarkers of drug‐induced liver injury in healthy human volunteers^1^

**DOI:** 10.1111/bcp.16371

**Published:** 2024-12-15

**Authors:** Andrea L. Jorgensen, Samantha Korver, Amy Schofield, Lawrence Howell, Joanna I. Clarke, Lauren E. Walker, Nathalie Brillant, Chris E. P. Goldring, Munir Pirmohamed

**Affiliations:** ^1^ Department of Health Data Science, Institute of Population Health University of Liverpool Liverpool UK; ^2^ Centre for Drug Safety Science, Department of Pharmacology and Therapeutics, Molecular & Clinical Pharmacology, Institute of Systems, Molecular and Integrative Biology University of Liverpool Liverpool UK

**Keywords:** biomarker, drug‐induced liver injury, qualification, validation

## Abstract

**Aims:**

The potential of mechanistic biomarkers to improve prediction of drug‐induced liver injury (DILI) and hepatic regeneration is widely acknowledged. We sought to determine reference intervals for new biomarkers of DILI and regeneration, as well as to characterize their natural variability and impact of diurnal variation.

**Methods:**

Serum samples from 227 healthy volunteers were recruited as part of a cross‐sectional study; of these, 25 subjects had weekly serial sampling over 3 weeks, while 23 had intensive blood sampling over a 24h period. Alanine aminotransferase (ALT), MicroRNA‐122 (miR‐122), High Mobility Group Box‐1 (HMGB1), total Keratin‐18 (K18), caspase‐cleaved Keratin‐18 (ccK18), Glutamate Dehydrogenase (GLDH) and Macrophage Colony‐Stimulating Factor‐1 (CSF‐1) were assayed.

**Results:**

Reference intervals were established for each biomarker based on the 97.5% quantile (90% CI) following the assessment of fixed effects in univariate and multivariable models. Intra‐individual variability was found to be non‐significant, and there was no significant impact of diurnal variation.

**Conclusion:**

Reference intervals for novel DILI biomarkers have been described. An upper limit of a reference range might represent the most appropriate mechanism to utilize these data. These data can now be used to interpret data from exploratory clinical DILI studies and to assist their further qualification as required by regulatory authorities.

**What is already known about this subject**

- Potential for novel biomarkers to improve prediction of drug‐induced liver injury (DILI) is widely acknowledged.
- It is necessary that reference intervals for these biomarkers are defined in several healthy human populations.

**What this study adds**

- Our study reports reference intervals for novel biomarkers of DILI in healthy volunteers.
- We report a reference interval for biomarker HGMB1 in healthy volunteers.
- Data will be a useful point of reference when interpreting exploratory clinical DILI studies.
- Data can also be used when seeking biomarker qualification by regulatory authorities.

## 1 INTRODUCTION

Drug‐induced liver injury (DILI) represents a major cause of patient morbidity and mortality and is a serious concern for patients, clinicians and the pharmaceutical industry.^1^, ^2^ It accounts for over half of the acute liver failure cases observed in Western countries and is a leading cause of withdrawal postmarketing.^1^, ^2^ Assessment of liver injury and repair has to date relied on a small number of blood‐based markers. These include serum alanine and aspartate aminotransferase activity (ALT and AST, respectively) as indicators of hepatocellular injury; alkaline phosphatase (ALP), γ‐glutamyl transferase (GGT) and total bilirubin (TBIL) for assessment of cholestatic injury; prothrombin time as a marker of liver synthetic capacity.^3^, ^4^ However, the lack of sensitivity and specificity of these tests, together with the fact that they provide little information about the mechanistic basis of liver injury, are significant limitations in using them for prediction and understanding DILI. Furthermore, elevation of these markers can be a result of disease/injury in nonhepatic sites,^5^ and the markers are of limited prognostic value.

A number of new biomarkers have shown promise in exploratory studies and preliminary evaluations, using samples from patients with liver injury during clinical trials and clinical practice.^5^, ^6^, ^7^, ^8^, ^9^, ^10^ These include biomarkers that provide enhanced hepatic specificity such as microRNA‐122 (miR‐122),^11^ biomarkers that can be used to monitor apoptotic‐necrotic dynamics such as keratin‐18 (K18), caspase‐cleaved K18 (ccK18)^9^, ^10^ and high mobility group box‐1 (HMGB1), mitochondrial dysfunction (glutamate dehydrogenase; GLDH),^8^, ^9^ and proteins that promote hepatic regeneration such as macrophage colony‐stimulating factor (CSF‐1). Both miR‐122 and GLDH are potential liver‐specific alternatives to ALT, having performed well as biomarkers for hepatocellular injury, particularly in patients with DILI resulting from paracetamol overdose^12^, ^13^, ^14^. The measurement of apoptotic *vs*. necrotic cell death using circulating K18 levels has also provided insights into the mechanistic basis of DILI in man.^8^, ^15^ Recent work has demonstrated the potential utility of these novel biomarkers, with a 3‐biomarker multivariate linear regression model achieving near perfect separation between patients with liver damage following paracetamol overdose and healthy controls (receiver operator characteristic, area under the curve = 0.99).^10^ Furthermore, this 3‐biomarker model confirmed strong predictability and specificity when comparing patients with liver damage to patient cohorts with muscle, pancreas, gastrointestinal and kidney damage (receiver operator characteristic, area under the curve = 0.98).^10^

Based on these observations, there has been significant interest and investment by biomarker consortia and DILI researcher networks to further develop and qualify novel DILI biomarker candidates that can add value, including mechanistic understanding, to current assessment methods. Importantly, these candidate biomarkers have been underpinned by supportive preclinical science^9^, ^16^, ^17^ and more recently, letters of support from regulatory authorities have backed the continued development and qualification of these candidate biomarkers across the spectrum of DILI observed in man.^13^, ^14^

Reference intervals in healthy volunteers were first reported by Church *et al*. as part of a larger investigation into the aforementioned biomarkers as candidate biomarkers for DILI.^9^ K18, ccK18 and miR‐122 levels in many healthy volunteers were found to be below the lower limit of quantification. Furthermore, miR‐122 demonstrated high inter‐ and intrasubject variability, which raised questions about its application and interpretation as a candidate biomarker for DILI. Given the potential utility of these biomarkers, it is important that reference intervals are defined in several healthy human populations, to aid further qualification of new biomarkers of liver injury or regeneration, so they can be appropriately interpreted in patients with DILI. As such, the aim of this current investigation was to establish reference intervals for miR122, GLDH, K18, ccK18, HMGB1 and CSF‐1, in a healthy human volunteer cohort and to assess the impact of inter‐ and intraindividual variation, and diurnal variation.

## 2 METHODS

### 2.1 Study population and ethical approval

To determine the degree of intra‐ and interindividual variation, and diurnal variation, of the biomarkers, 3 prospective studies were conducted. Studies 1 and 2 considered the impact of inter‐ and intraindividual variation, respectively, while study 3 assessed the impact of diurnal variation. All investigations were conducted in compliance with the guidelines of the Declaration of Helsinki and ICH‐GCP and reported to the Standards for Reporting Diagnostic Accuracy,^18^, ^19^ and all participants gave written informed consent. For studies 1 and 2, ethical approval was from the North West Centre of Research Ethics Committee, UK. Ethical approval for study 3 was initially granted by the North West Research Ethics Committee Manchester Central (12/NW/0374). Following relocation of the study to the San Antonio site in the USA, approval was granted by the institutional review board (LLC 0327/014).

Approval covered collection of blood from volunteers up to a maximum of 400 mL over a 24‐h sampling period. Approval was given for the analysis for any potentially novel biomarker and/or inflammatory marker. For study 3, additional restrictions included diet and concurrent medications. All subjects were provided with standardized meals and snacks at set times during the admission to the Phase I units. Any prescription/nonprescription drug, vitamin or dietary supplement was forbidden within 2 weeks of admission to the clinical unit. Herbal supplements were discontinued at least 4 weeks prior to admission.

For all 3 studies, adults (aged ≥18 years) were recruited unless they had the following exclusion criteria: a positive test for HIV, hepatitis B or hepatitis C viral infections, a medical intervention performed within 3 months of study enrolment, a positive pregnancy test, or unwillingness to refrain from illicit drug/alcohol/tobacco, paracetamol use or strenuous exercise during the study and for 7 days prior to sample collection. Full details of sample collection protocols, serum preparation and samples handling/storage, are described in the Supplementary Material 2: Methods.

### 2.2 Assessment of inter‐ and intraindividual variation (studies 1 and 2)

Healthy volunteers (*n* = 227) were recruited between November 2010 and October 2014, and in July 2018 in Liverpool, UK. Blood samples were taken between 08.00 and 12.00 on day 1 of recruitment for all subjects as part of study 1. A subset of 25 out of the 227 individuals participated in study 2 to determine intraindividual variability. These 25 subjects returned for 2 further visits at weekly intervals for blood sample collection.

### 2.3 Assessment of diurnal variation (study 3)

For study 3, 23 volunteers were recruited, initially from the ICON Development Solutions Clinical Unit located on the Manchester Royal Infirmary hospital site (*n* = 4), and subsequently from the ICON Phase 1 unit, San Antonio, Texas, USA (*n* = 20). Only the biomarkers miR‐122, HMGB1, ccK18 and K18 were measured in study 3.

On the day before sampling, subjects were admitted to the unit at 13.00 h and were provided with a standardized meal at 18.00 h, followed by a standardized snack at 21.00 h, following which the subjects underwent a 10‐h overnight fast. On the day of the study, blood samples were taken at 07:00 h and then every 3 h over a 24‐h period between 07:00 h and 22:00 h, and then again at 00:00 h on day 1 and 04:00 h on day 2. Subjects were discharged at 08.00 h on day 2.

### 2.4 Sample size calculation

The International Federation of Clinical Chemistry (IFCC) recommends that samples from 120 or more individuals are required for the development of reference intervals for analytes.^20^ Most studies undertake bi‐daily sampling (morning and evening). This study was the first of its kind to examine 3‐hourly samples across a complete 24‐h period. As such, a sample size calculation could not be performed based on published studies. An estimation of 23 individuals was undertaken with a view to increasing recruitment should it have been felt necessary.

### 2.5 Biomarker measurements

miR‐122, ccK18 (caspase cleaved K18), K18 (total K18), GLDH and CSF‐1 were measured as previously reported in literature (Supplementary Material 2: Methods).^9^ HMGB1 was measured using a commercially available ELISA kit (Supplementary Material 2: Methods). For each sample, biomarkers were measured in duplicate. The miR‐122 concentration was expressed with reference to let‐7d as the internal microRNA normalizer, and expressed as copies/μL. Investigators measuring the biomarkers were blinded to the details of the volunteers. All assays or reagents were commercially available. Assay development and validation has been described previously. When appropriate, assay validation was based on Clinical and Laboratory Standards Institute and IFCC guidelines. Further detailed protocols are provided within the Supplementary Material 2: Methods.

### 2.6 Statistical analysis and reference interval determination

Biomarker concentrations from the day 1 samples produced by each participant in study 1 were used to calculate the overall reference intervals using both Meso Scale Discovery and Luminex‐based analytical approaches alongside R version 3.5.2.^21^

The distributions of the biomarkers were investigated to determine whether they required a log or square root transformation. The appropriate transformation was applied, if required, to the biomarker values to approximate a Gaussian distribution, fulfilling the normality assumption of subsequent analyses. Outliers were identified to the transformed biomarker values and removed according to Tukey's method.^22^ For each biomarker in turn, a linear model was fitted with each of the factors: sex, age, height, weight, body mass index (BMI) and ethnicity included as fixed effects, 1 at a time to assess the need for partitioning of the reference population by the factor. Every possible combination of fixed effects found significant in the univariate models (Pr[>χ^2^] < 0.05) were combined in a multivariable model and the optimal model determined with reference to Akaike information criterion. If any of the fixed effects were significant, reference ranges per strata were calculated using quantile regression.

Where quantiles were not found to vary significantly with any fixed factors, the 2.5th and 97.5th quantiles were referred to in order to calculate the 95% reference intervals for each biomarker. The 90% confidence intervals (CIs) for the upper limit of the reference interval were also calculated. Where quantiles were found to vary significantly with fixed factors, we report the predicted values for the 2.5th and 97.5th quantiles, as obtained from the fitted quantile regression model, for particular strata, together with 90% CIs for the upper limit. Choice of boundaries for the fixed factor strata were determined by clinical expertise.

To explore intraindividual variability, for the 25 participants in study 2, a linear mixed model was fitted for each biomarker in turn, including the fixed effects identified as significant in study 1, together with a random intercept term to represent each individual participant. The linear mixed model was then compared to a linear model including the fixed effects only, using a likelihood ratio test. Where there were no significant fixed effects identified in study 1, a fixed intercept linear model was compared to a linear mixed model with random intercept.

To explore if there was a diurnal pattern in the level of any of the biomarkers under investigation, Study 3 data were analysed again using a linear mixed model approach with biomarker data transformed as necessary to achieve a Gaussian distribution, and outliers were identified and removed according to Tukey's method.^22^ Fixed effects found significant in the analysis of Study 1 and 2 were included, with the exception of BMI, which was unavailable for these patients, as well as a random effect term for the time point at which a measurement was taken from individual subjects. This random effect term was tested for significance using a likelihood ratio test comparing models both with and without the random effect.

## 3 RESULTS

### 3.1 Determination of reference intervals in a cross‐sectional analysis and the impact of intra‐ and intersubject variation

**FIGURE 1:**
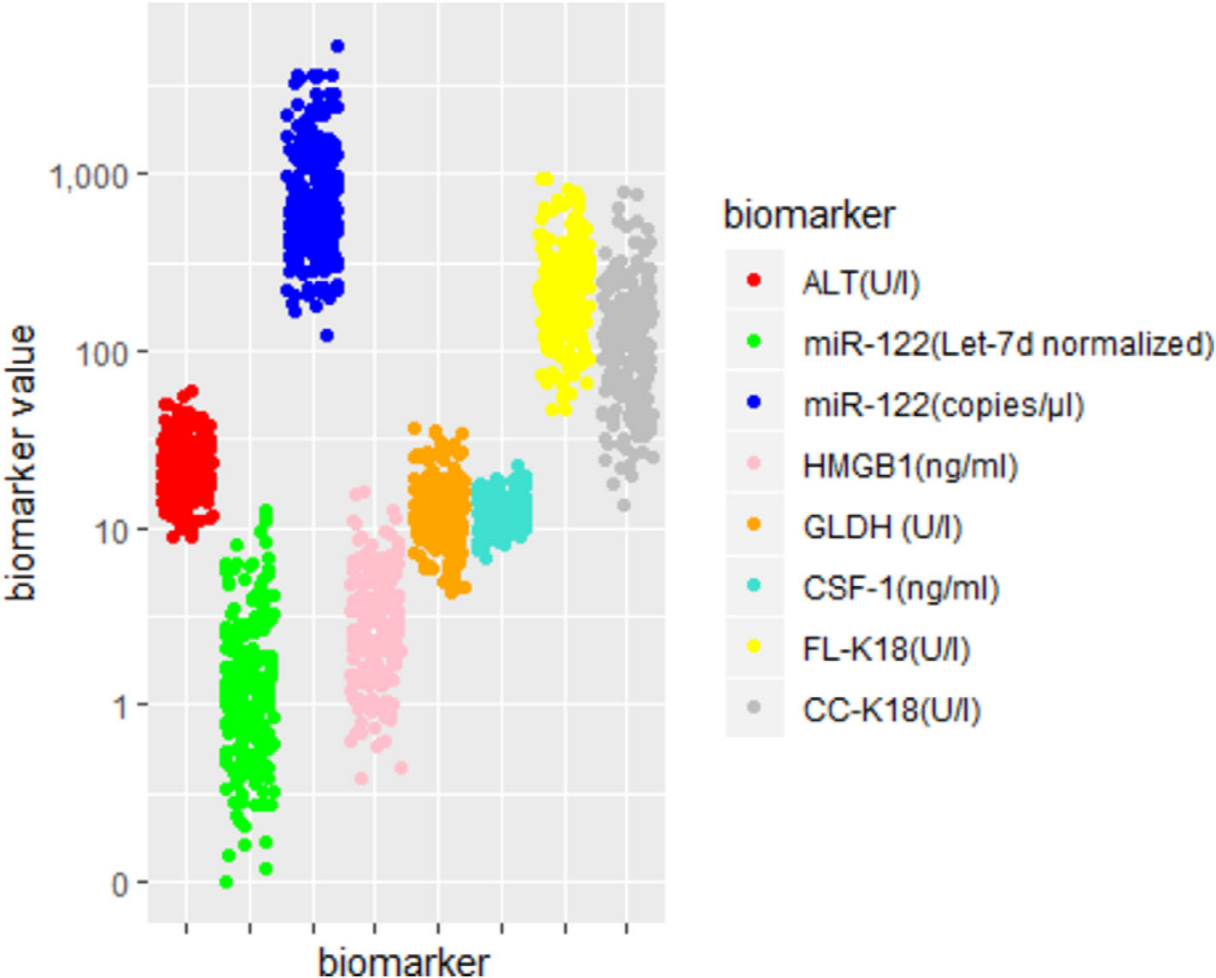
Scatterplot of biomarker values from study 1. ALT, alanine aminotransferase; BMI, body mass index; ccK18, caspase‐cleaved keratin‐18; CI, confidence interval; CSF‐1, colony‐stimulating factor‐1; FL‐K18, full‐legth keratin‐18; GLDH, glutamate dehydrogenase; HGMB1, high mobility group box‐1; K18, keratin‐18.

The natural interindividual variation of candidate biomarkers was explored in 227 healthy volunteers. The demographics of the volunteers recruited to each study are described in Table 1. All assays used were technically validated when appropriate, sample processing was standardized and the findings and guidance for the use of each assay and sample storage is summarized in the Supplementary Material 2: Methods. The range and spread of the biomarker values are depicted graphically in Figure 1. The outcome of our statistical analysis demonstrated that there were no significant fixed effects in the univariate model for ccK18, K18 and CSF‐1. However, sex and BMI were significant fixed effects in the multivariable model for ALT and miR‐122 (let‐7d normalized). Sex was also a significant fixed effect for miR122 (copies/μL), whilst BMI was a significant fixed effect for GLDH, and age was a significant fixed effect for HMGB1. A summary of the estimates of fixed effects for each of the biomarkers is shown in Table S2.

**TABLE 1:** Demographic data of healthy volunteers participating in each study^1^.

Characteristic	Study 1 + 2 (*n =* 227)	Study 3 (*n =* 23)
Sex (male: female)	144: 81	14: 9
Ethnicity (% Caucasian)	87	70
(% Black)	1	30
(% Asian)	10	0
(% Other)	2	0
Age (years), median (IQR)	32 (19–64)	38 (19–46)
Body mass index (kg/m^2^), median (IQR)	24 (18–44)	NA

Abbreviation: IQR, interquartile range.

^1^ Sex missing for 2 participants; ethnicity missing for 3 participants; body mass index missing for 2 participants.

Table 2 summarizes the 2.5th, 50th and 97.5th quantiles (together with 90% CIs) with the 97.5th quantile representing the upper limit of normal for this reference population. For the biomarkers with significant effects, each quantile was calculated for various strata. Sex was stratified as male and female, BMI was stratified according to current UK National Health Service guidelines (underweight <18.5, healthy 18.5–24.9, overweight 25–29.9 and obese >30 kg/m^2^), whilst age was stratified according to the changes in variability observed in the data (18–44, 45–60 and 60+ years).

**TABLE 2:** Reference intervals for biomarkers of drug‐induced liver injury and regeneration.

Biomarker	Grouping	2.5th quantile (90% CI)	50th quantile (90% CI)	97.5th quantile (90% CI)
**ALT (U/I)**	Female BMI Healthy	8.65 (4.82, 15.52)	16.53 (9.12, 29.94)	35.53 (18.99, 66.46)
	Female BMI Overweight	10.06 (5.22, 19.38)	19.02 (9.77, 37.01)	40.43 (19.97, 81.82)
	Female BMI Obese	12.24 (5.80, 25.82)	22.80 (10.68, 48.70)	47.77 (21.32, 107.06)
	Male BMI Healthy	12.93 (6.56, 25.45)	22.30 (10.89, 45.64)	45.48 (21.62, 95.66)
	Male BMI Overweight	15.04 (7.12, 31.78)	25.66 (11.67, 56.42)	51.75 (22.74, 117.76)
	Male BMI Obese	18.30 (7.91, 42.34)	30.76 (12.75, 74.23)	61.15 (24.27, 154.09)
**miR‐122** **(let‐7d normalized)**	Female BMI Healthy	0.17 (0.02, 1.16)	1.02 (0.19, 5.53)	8.64 (1.70, 43.81)
	Female BMI Overweight	0.15 (0.02, 1.32)	1.33 (0.20, 8.86)	8.32 (1.36, 50.92)
	Female BMI Obese	0.13 (0.01, 1.57)	1.87 (0.22, 16.29)	7.93 (1.02, 61.86)
	Male BMI Healthy	0.28 (0.03, 2.63)	1.35 (0.18, 9.99)	9.73 (1.41, 67.12)
	Male BMI Overweight	0.25 (0.02, 3.01)	1.76 (0.19, 16.00)	9.38 (1.13, 78.02)
	Male BMI Obese	0.22 (0.01, 3.58)	2.47 (0.21, 29.44)	8.94 (0.84, 94.78)
**miR122** **(copies/μL)**	Female	204.28 (187.41, 222.67)	521.87 (451.53, 603.17)	2850.20 (2407.75, 3373.95)
	Male	233.89 (179.16, 305.34)	873.68 (593.54, 1286.04)	4103.94 (2677.39, 6290.59)
**HMGB1** **(ng/mL)**	Age 30	0.59 (0.24, 1.46)	2.60 (1.26, 5.39)	9.45 (5.69, 15.71)
	Age 45	0.55 (0.18, 1.64)	2.27 (0.93, 5.54)	7.97 (4.27, 14.86)
	Age 60	0.51 (0.14, 1.84)	1.97 (0.69, 5.68)	6.72 (3.21, 14.06)
**ccK18** **(U/I)**	No grouping	24.62 (17.72, 27.08)	120.69 (108.23, 132.58)	544.56 (416.15, 785.22)
**FL‐K18** **(U/I)**	No grouping	63.63 (46.89, 70.73)	205.20 (186.75, 227.39)	757.80 (702.45, 927.99)
**GLDH** **(U/I)**	BMI Healthy	4.71 (2.76, 8.04)	11.63 (6.24, 21.67)	31.24 (16.26, 60.00)
	BMI Overweight	6.21 (3.42, 11.27)	12.62 (6.28, 25.36)	28.38 (13.73, 58.66)
	BMI Obese	8.87 (4.51, 17.44)	14.03 (6.33, 31.08)	25.06 (11.02, 56.98)
**CSF‐1** **(ng/mL)**	No grouping	7.96 (7.60, 8.62)	12.18 (11.81, 12.47)	18.84 (18.11, 19.87)

Abbreviations: ALT, alanine aminotransferase; BMI, body mass index; ccK18, caspase‐cleaved keratin‐18; CI, confidence interval; CSF‐1, colony‐stimulating factor‐1; FL‐K18, full‐legth keratin‐18; GLDH, glutamate dehydrogenase; HGMB1, high mobility group box‐1; K18, keratin‐18.

We also assessed intraindividual variability using data from study 2, by including a random intercept term to linear models including significant fixed effects. This did not improve the model fit for any of the biomarkers, suggesting that there was no significant intraindividual variability.

### 3.2 Assessment of the impact of diurnal variation on observed biomarker values in individual subjects

**FIGURE 2:**
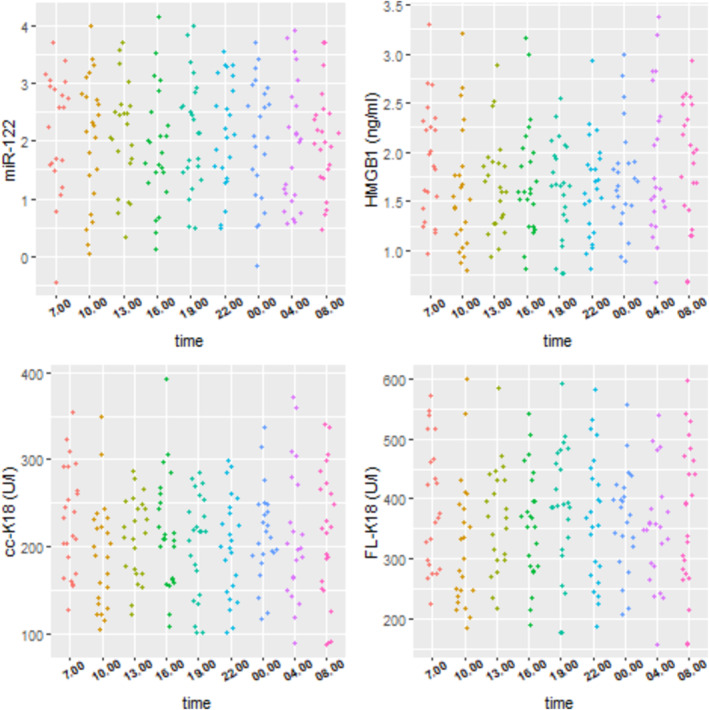
Scatterplots of biomarker values *vs*. time from study 3.

Patients with DILI can present clinically at any time of the day. Therefore, an assessment of the potential impact of diurnal variation is an important consideration for establishing reference intervals and to assist with the interpretation of biomarker data. Figure 2 demonstrates the observed biomarker values qualified from individual subjects across an intensive 24‐h sampling period. No significant time‐random effect was identified for any of the biomarkers.

## 4 DISCUSSION

Exploratory studies of DILI, from both clinical trials and clinical practice, have demonstrated the significant potential and clinical validity of novel mechanism‐based biomarkers of liver injury and regeneration to complement current liver function tests such as transaminases. However, the correct interpretation of these novel tests is dependent on the determination of reference ranges in healthy volunteers. In this study, we report comprehensive reference intervals for novel biomarkers of DILI and hepatic regeneration, derived from prospectively designed human volunteer cohorts using standardized protocols and commercially available and technically validated assays.

Our findings indicate there was no significant intra‐ or interindividual variation in any of the biomarkers in serum investigated across the study. This is particularly significant in regards to miR‐122 and GLDH, where previously reported reference intervals in healthy participants have been beset by problems of both inter‐ and intraindividual variability.^9^ For example, for miR‐122, we found the intraindividual variability to be 41%, compared with 94% reported by Church *et al*.^9^ In this study, BMI (GLDH) and sex (miR‐122) were determined as significant fixed effects from linear modelling, and reference intervals for BMI groupings and sex were established for each novel biomarker, respectively. As the expression of both GLDH and miR‐122 are enriched in the liver, factors such as visceral fat and sex that influence basal liver function may affect high inter‐ and intraindividual variability in their serum levels. Therefore, establishing reference intervals based on significant fixed effects can reduce intra or interindividual variation in biomarker levels as we have demonstrated. The current clinical chemistry standard for assessing hepatocellular injury, ALT, is not liver‐specific and can be elevated in nonliver disease such as cardiac injury^23^, ^24^, ^25^ and skeletal muscle damage.^26^ The shortcomings of ALT as a biomarker have been widely discussed.^27^ It is therefore important that future studies evaluate the potential role of liver‐enriched biomarkers, providing an early indication of hepatocyte damage in acute settings and enhanced liver specificity over current markers.^15^

Healthy reference intervals for K18 and ccK18 have been previously reported in both small (17 volunteers) and medium (100 and 150 volunteers) sized control groups.^28^, ^29^, ^30^ The mean ccK18 level reported in these studies was consistent with our findings.^28^, ^29^, ^30^ However, variability in mean K18 levels across studies was high, suggesting the ubiquitous expression of K18 may have a bigger influence on circulating K18 levels in healthy controls than first thought. The level of K18 and ccK18 present in plasma or serum can be reflective of the degree of hepatocellular necrotic and/or apoptotic injury and although both biomarkers have successfully identified such injury, variability in K18 and ccK18 levels in disease states is also relatively high.^28^, ^29^, ^30^, ^31^

Literature has reported healthy reference intervals for CSF‐1 in small control groups (20 and 50 volunteers) whereas we are the first to establish a healthy reference interval for a relatively large number of healthy controls.^32^, ^33^ In these studies, CSF‐1 was not identified as a serum biomarker for head and neck squamous cell cancer, yet CSF‐1 was a sensitive diagnostic marker for patients with pancreatic and ampullary cancer, with increased diagnostic sensitivity and specificity in comparison to other measured markers GM‐CSF and IL‐3.^29^, ^30^ The mean CSF‐1 serum level in our study was 12.18 ng/mL compared to a previously reported mean of 0.251 ng/mL in 50 healthy volunteers.^30^ These concentrations suggest that CSF‐1, and potentially other novel biomarkers investigated in this study, may be significantly lost during the clotting process that takes place when collecting serum from whole blood. This has been demonstrated in a study screening for biomarkers using mass spectrometry, which showed that levels can vary according to whether the blood tubes contain an anticoagulant.^34^

Our dataset represents a significant step forward in development of new DILI biomarkers that can be used to support current tests and should contribute to the evaluation of the potential impact of variability within a population. It is important to note that we do not yet advocate that these biomarkers will replace currently measured and accepted measures of liver function. Rather, they can be used to further complement existing parameters and fill current knowledge gaps such as mechanistic insights and the need for increased organ specificity and prognostic value.

In addition to variability in both sample processing and natural biological processes, diurnal variation is an important source of systematic variability, being consistent in nature as opposed to the random fluctuations around the mean inherent to steady‐state periods. This is clinically relevant given that patients can present to the hospital emergency room at any time across a 24‐h period following emergencies such as automatic positive airway pressure overdose, or indeed any form of drug‐induced acute liver failure. Our data demonstrate that there was no significant diurnal variation amongst any of the biomarkers in the panel measured. To date, there have been limited biomarker studies investigating the impact of diurnal or circadian variation, and no studies reported, to the best of our knowledge, that included intensive sampling over a 24‐h period as we have undertaken. In a report investigating prognostic biomarkers for osteoarthritis, circadian variation in hyaluronan, keratin sulfate and cartilage oligomatrix protein was reported,^35^ but this study was limited by a small number of sampling time points spanning >24 h. In our study, the finding that there was no significant diurnal impact, across an intensive 24‐h multiple sampling period in the biomarkers we measured, should be relevant for the further development and clinical uptake of these DILI biomarkers.

Since no single objective DILI biomarker has yet been qualified, current practice places a heavy burden on the interpretation of ALT values in detecting impending hepatocellular injury. However, little consensus exists as to whether or not to use a fold‐change based on a laboratory reference range or an individual's own baseline values when assessing potential liver injury. This of course may be context‐dependent, in terms of whether it is in the *real world*, or in a controlled clinical trial setting. Nevertheless, based on our observations that there is low intra‐ and interindividual variability for our panel of biomarkers, we have described our reference intervals in terms of the 97.5% quantile. We recommend that the upper limit of a reference range for these new biomarkers might represent the most appropriate way to use these data. This may be particularly important for the application of such a new biomarker class in the real‐world setting, when patients often present with established liver injury, or their own baseline cannot be established.

Our study has some limitations. Our biomarker list is not exhaustive but is based on biomarkers that have some evidence of clinical utility, or that support enhanced mechanistic insights.^15^ The median age of the volunteers was 32 years and only 22 out of 227 were older than 50 years. Therefore, there is a lack of a representation of older individuals within our group. This may be particularly important in the context of idiosyncratic DILI where the average reported ages from international registries are between 48 and 55 years.^36^ However, despite this, the demographics accurately reflect the population who typically take an paracetamol overdose, which still remains a significant clinical problem. Furthermore, age was determined to be a significant reason for interindividual variability for only 1 of the biomarkers investigated, HMGB1. Despite exceeding the requirements set by the IFCC for the minimum number of individuals required for the development of reference intervals for analytes, we did not include an external validation cohort to replicate our findings. However, we note that the reference intervals for HMGB1 are consistent with a large study of healthy volunteers (*n* = 626).^37^ For ALT, the reference intervals defined in our study are consistent with those previously described.^38^ Moreover, in our study, we noted a significant impact of sex and BMI on observed ALT values, which is in line with other studies.^38^ In addition, our healthy volunteer population was primarily Caucasian, with a small number of African American participants reported in Study 3. We did not identify any ethnic variability in reference range intervals between Caucasian and African American participants in Study 3, which is expected due to the small population. However, we are aware that this is a limitation of the study as basal levels of biological markers can differ between ethnicities.

In conclusion, for DILI, there is an unmet need to develop biomarkers with improved specificity and sensitivity that predict the onset and progression of pathology and response to treatment. The assessment of clinical validity of a biomarker is central to its development; this has been previously demonstrated with the panel of biomarkers under investigation here.^5^, ^9^, ^10^, ^11^ However, correct interpretation of biomarker data is dependent on the comprehensive and accurate attainment of reference biomarker values in the healthy state and an understanding of factors that affect variability. The data from the present study provides reference intervals for novel biomarkers of DILI and regeneration in healthy volunteers, as well as robust assessments of intra‐ and interindividual variability, and diurnal variation. These data can now be used to compare these potential biomarkers against the growing repository of literature that is evaluating the utility of these in DILI patients, which will be of importance in the pathway towards biomarker qualification.

## AUTHOR CONTRIBUTIONS

A.L.J. and M.P. wrote the article; A.S., J.I.C., L.E.W., N.B., S.K., A.L.J., C.E.P.G., L.H. and M.P. performed critical manuscript revisions; L.E.W. and M.P. designed research and wrote study protocols; L.E.W. and M.P. coordinated volunteer recruitment; A.S., J.I.C., L.E.W., L.H. and N.B. performed biomarker measurements; A.L.J. analysed data; M.P. provided reagents and infrastructural support.

## CONFLICT OF INTEREST STATEMENT

M.P. currently receives partnership funding, paid to the University of Liverpool, for the following: MRC Clinical Pharmacology Training Scheme (cofunded by MRC and Roche, UCB, Eli Lilly and Novartis), and the MRC Medicines Development Fellowship Scheme (cofunded by MRC and GSK, AZ, Optum and Hammersmith Medicines Research). He has developed an HLA genotyping panel with MC Diagnostics but does not benefit financially from this. He is part of the IMI Consortium ARDAT (www.ardat.org); none of these of funding sources have been used for the current research.

## Supporting information


**Table S1.** Validation parameters and assay platforms for measured biomarkers.
**Table S2.** Fixed effects from final linear models.

## Data Availability

The data that support the findings of this study are available on request from the corresponding author. The data are not publicly available due to privacy or ethical restrictions.
