# Supplementary material for: Establishing reference ranges for circulating biomarkers of drug‐induced liver injury in healthy human volunteers
Source: Br J Clin Pharmacol. 2024 Dec 15;91(5):1361–9. doi: 10.1111/bcp.16371 (PMC12035588; doi:10.1111/bcp.16371)
Supplement: Supplementary file 1 — Table S1. Validation parameters and assay platforms for measured biomarkers. Table S2. Fixed effects from final linear models. [file BCP-91-1361-s001.docx]

**SUPPLEMENTARY APPENDIX**

**Establishing reference ranges for circulating biomarkers of Drug-Induced Liver Injury in healthy human volunteers**

Andrea L Jorgensen^1a^, Samantha Korver^2a^, Amy Schofield^2^, Lawrence Howell^2^, Joanna I Clarke^2^, Lauren E Walker^2^, Nathalie Brillant^2^, Chris E. P. Goldring^2^, Munir Pirmohamed^2^

^1^Department of Health Data Science, Institute of Population Health, University of Liverpool, Liverpool, UK.

^2^Centre for Drug Safety Science, Department of Pharmacology and Therapeutics, Molecular & Clinical Pharmacology, Institute of Systems, Molecular and Integrative Biology, University of Liverpool, Liverpool, UK.

^a^Joint first authors

**Corresponding author:**

Prof. Sir Munir Pirmohamed (munirp@liverpool.ac.uk)

The authors confirm that the PI for this paper is Prof. Sir Munir Pirmohamed and he had direct clinical responsibility for patients.

**SUPPLEMENTARY MATERIAL 1; STATEMENT OF REFERENCE TO THE ORIGINAL RESEARH PAPER**

As part of an investigation by the University of Liverpool into research misconduct by a University of Liverpool academic, Dr Daniel J Antoine, the senior author of the original paper, the co-authors of the paper identified evidence of data falsification. This investigation was upheld, and the original paper was withdrawn from the Journal of Hepatology. However, there was no question as to the authenticity of the original samples and it was confirmed that these samples were obtained under the appropriate ethical procedures. As such, the co-authors in addition to two new co-authors (SK and CEPG), repeated the analysis and rewrote the original manuscript. For re-analysis, none of the original sample aliquots were used. Aliquots that had been stored at -150^0^C, not previously freeze-thawed and not previously used in the original manuscript were analysed to maintain not only sample integrity but also minimise sample degradation. None of the original data from the original manuscript has been used.

**SUPPLEMENTARY MATERIAL 2; METHODS**

**Sample collection, handling and serum preparation**

Blood collection was prospectively collected for this investigation and serum preparation and storage was standardized across all studies. Ten (10) ml of blood was collected in plain red top vacuum blood collection tubes twice. To reduce the potential of sample matrix to contribute to observed variability in biomarker values, only one matrix was prepared and analysed for biomarker measurements. Briefly, serum was prepared after blood being able to clot at room temperature for 10 minutes and then undergoing centrifugation at 1300 xg for 15 minutes. Serum was removed and stored in 500 uL ml aliquots at -80°C. All serum samples were prepared within 2 hours of the blood sample being obtained. All serum samples were shipped on dry ice by overnight courier, handled, stored and processed at one central site for biomarker measurements to minimise the impact of multiple site handling contributing to variability of observed measurements. Samples were thawed on ice over 1-2 hours prior to biomarker analysis. All samples were assayed in one batch per biomarker at the completion of the study and with investigators performing biomarker assays being blinded to any clinical data and sample timings.

**miRNA isolation and PCR set up and detailed protocol for miRNA quantification**

For ELISAs or activity assays **(Supplementary Table 1),** samples were immediately utilised in the required assay according to the manufacturer’s protocol and underwent no further extraction. Serum present miRNAs were isolated following manufactures protocols (QIAGEN) for miRNeasy kits from 40 μl of serum diluted to 200 μl with nuclease-free water. miRNA isolation and PCR set up was conducted in an automated way using the QIAcube and QIAgility instruments. Seven hundred (700) µl of QIAzol reagent were added to serum samples, mixed and incubated for 5 minutes before the addition of 140 µl chloroform. Samples were then mixed vigorously and centrifuged at 12,000 g and 4 °C for 15 mins. 350 µl of the upper aqueous phase were transferred to fresh micro tubes for automated extraction and purification; this included the addition of 350 µl 70 % ethanol, application to miRNeasy mini spin columns and centrifuged at >8,000 g for 15 seconds. The flow-through was added to 450 µl of 100 % ethanol, samples applied to RNeasy Min Elute columns and centrifuged at >8,000 g for 15 seconds. The elution was then purified by washing with various buffers before a final 80 % ethanol wash. The columns were then dried by centrifugation and the small RNA fraction was eluted in 14 µl of nuclease-free water before storing at -80 °C.

Specific miRNA levels were measured using TaqMan-based quantitative polymerase chain reaction (qPCR). The small RNA elutes were reverse transcribed using specific stem-loop reverse transcription (RT) primers (Life Technologies, Carlsbad, USA) for each target miRNA species (hsa-miR-122-5p, hsa-let-7d-5p) in accordance with the manufacturer’s instructions. Two (2) µl purified miRNA were used to synthesise cDNA with a total reaction volume of 15 µl via thermal cycling; 30 minutes at 16 °C, 30 minutes at 42 °C, 5 minutes at 85 °C and held at 4 °C.

Then, 6.3 µl of cDNA from the RT were used, in duplicate, with the use of corresponding specific PCR primers (Life Technologies) in a total reaction volume of 20 µl. After undergoing thermal cycling of 2 minutes at 50 °C, 10 minutes at 95 °C and 50 cycles of 15 seconds at 95 °C and 60 seconds at 60 °C, levels of miRNA were measured by the fluorescent signal produced from the TaqMan assay probes. Relative as well as absolute quantification were evaluated; miR-122 levels were either normalised to the level of endogenous miRNA: let-7d (AGAGGUAGUAGGUUGCAUAGUU) or miR-122 copy numbers were calculated through the use of a standard curve. Additionally, the standard curves were included on each individual qPCR plate in order to ensure the accuracy of each step within the protocol. Validation of assays was performed based on CSLI guidelines. Intra-assay precision was assessed by measuring 5 samples individual on one day and intra-assay precision was assessed by measuring 1 sample on 5 independent days. Acceptable degrees of variation were prospectively set at <15% C.V. Dilutional linearity was assessed over 1:2 serial dilutions with acceptable degrees of recovery being prospectively set at 85 – 115%.

**Quantification of circulating protein biomarkers**

K18 and ccK18 (Peviva; M30 apoptosense and M65 classic), total HMGB1 (IBL) and CSF-1 (Mesoscale discovery) content was determined using commercially available assays. These kits have been readily used previously [[9](#_ENREF_9), [10](#_ENREF_10), [40-45](#_ENREF_40)] and were conducted as follows:


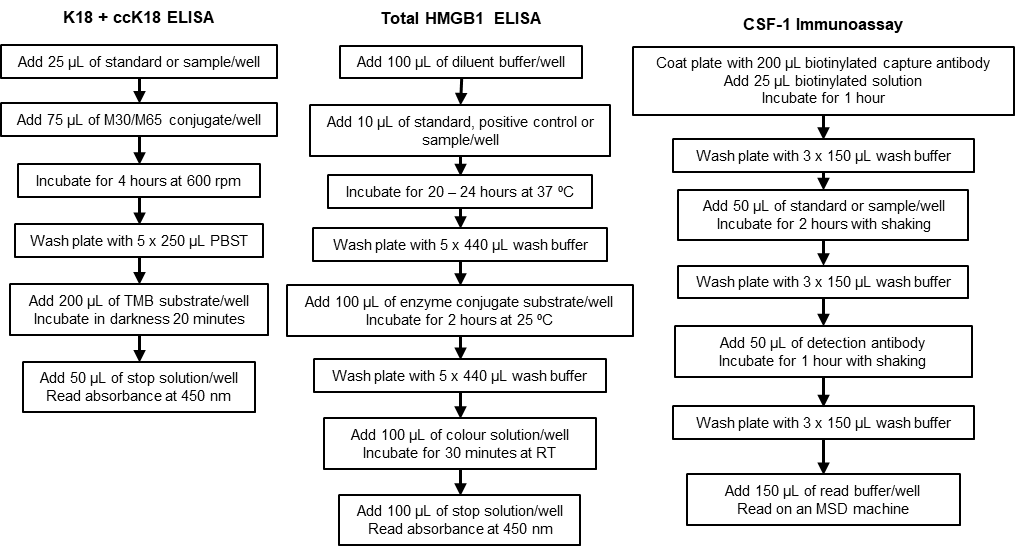


Prior to being assayed, thawed samples were centrifuged at 2,000 g for 1 min and the supernatant used. Biomarker content was detected by turnover of 3,3’,5,5’-tetramethylbenzidine (TMB) by a peroxidase-linked anti-analyte antibody at 450 nm using a Varioskan Flash machine (Thermo Scientific) and compared against an authentic recombinant standard. Validation of ELISAs were performed as per CSLI guidelines. Intra-assay precision was assessed by measuring 5 samples individual on one day and intra-assay precision was assessed by measuring 1 sample on 5 independent days. Acceptable degrees of variation were prospectively set at <15% C.V. Recovery was assayed against a sample spiked with recombinant protein at low and high clinical relevant concentrations (ccK18; 300 and 1000 U/l, FL-K18; 600 and 1200 U/l, HMGB1 10 and 50 ng/ml, CSF-1; 5 and 20 ng/ml). Acceptable degrees of variation were prospectively set between 85 – 115%. Dilutional linearity was assessed over 1:2 serial dilutions with acceptable degrees of recovery being prospectively set at 85 – 115%.

**Quantification of serum ALT and GLDH activity**


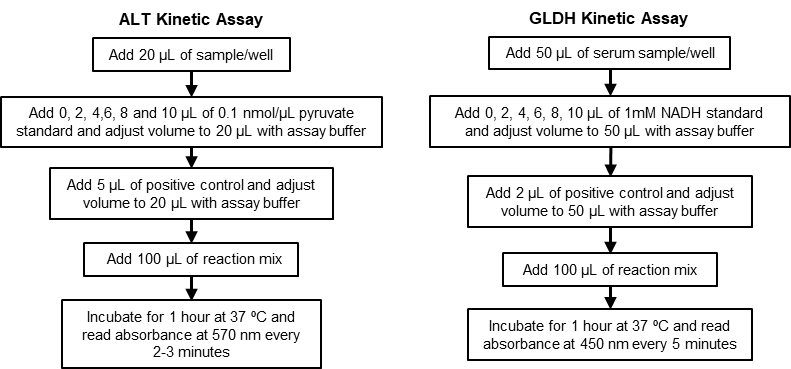
Serum ALT was determined by a photometric kinetic assay using Infinity^TM^ ALT (GPT) Liquid Stable Reagent (ThermoFisher Scientific) in accordance with the manufacturer’s instructions. Normal and abnormal Data TrolTM Control serum samples were ran as unknown samples to confirm quality control. ALT reagent was pre-warmed to 37°C prior to use and samples were assayed with a Varioskan Flash machine (Thermo Scientific). Serum glutamate dehydrogenase (GLDH) levels were determined using a commercial photometric kinetic assay Abcam (ab102527) in accordance with the manufacturer’s instructions. Intra-assay precision was assessed by measuring 5 samples individual on one day and intra-assay precision was assessed by measuring 1 sample on 5 independent days. Acceptable degrees of variation were prospectively set at <15% C.V. Dilutional linearity was assessed over 1:2 serial dilutions with acceptable degrees of recovery being prospectively set at 85 – 115%.

**Supplementary Table 1:** Validation parameters and assay platforms for measured biomarkers^1^

| **Biomarker and Assay platform** | **Sample matrix** | **Unit** | **LLoQ** | **ULoQ** | **Intra-assay**  **(% CV)** | **Inter-assay**  **(% CV)** | **Low spike recovery (%)** | **High spike recovery (%)** | **Dilutional linearity** | **Freeze/thaw stability cycles** |
| --- | --- | --- | --- | --- | --- | --- | --- | --- | --- | --- |
| ALT  Activity | S | U/l | 5 | 450 | 7.6 | 5.2 – 11.1 | ND | ND | 1:64 | 1 |
| CSF-1  ELISA | S | ng/ml | 0.2 | 120 | 10.1 | 6.3 – 13.1 | 91.1 (11.0) | 86.7 (12.1) | 1:16 | 3 |
| GLDH  Activity | S | mU/ml | 0.01 |  | ND | ND | ND | ND | ND | 1 |
| HMGB1  ELISA | S | ng/ml | 0.2 | 80 | 5.1 | 2.6 – 6.3 | 94.6 (8.9) | 84.0 (5.6) | 1:16 | 3 |
| ccK18  ELISA | S | U/l | 75 | 1000 | 5.5 | 3.2 – 8.7 | 106.2 (5.2) | 85.9 (11.8) | 1:32 | 3 |
| FL-K18  ELISA | S | U/l | 125 | 2000 | 6.5 | 5.5 – 8.5 | 100.7 (9.6) | 105.7 (12.1) | 1:32 | 3 |
| miR-12  qRT-PCR | S | copies/μl | 106 | 29325287 | 7.6 | 4.8 – 14.8 | ND | ND | 1:16 | 3 |

^1^Lower limit of quantification (LLoQ), upper limit of quantification (ULoQ), coefficient of variability (%CV), Alanine aminotransferase (ALT), Full length Keratin 18 (K18), caspase cleaved Keratin 18 (ccK18), glutamate dehydrogenase (GLDH), not determined (ND), High Mobility Group Box-1 (HMGB1), Colony Stimulating factor 1 (CSF-1), microRNA-122 (miR-122), quantitative reverse transcription real-time PCR (qRT-PCR), serum sample (S).

**Supplementary Table 2:** Fixed effects from final linear models

| **Biomarker** | **Covariate** | **Coefficient^1^** | **SE^2^** |
| --- | --- | --- | --- |
| ALT (U/I) | sex (male) | 0.262 | 0.050 |
|  | BMI (kg/m^2^) | 0.021 | 0.005 |
| miR-122 (Let-7d normalized) | sex (male) | 0.313 | 0.134 |
|  | BMI (kg/m^2^) | 0.037 | 0.015 |
| miR122 (copies/ µl) | sex (male) | 0.298 | 0.103 |
| HMGB1 (ng/ml) | age (years) | -0.013 | 0.005 |
| GLDH (U/I) | BMI (kg/m^2^) | 0.017 | 0.007 |

^2^Estimates are from fitting linear multiple regression model to log-transformed biomarkers
